# Cholinesterase Inhibitory Activities of Adamantyl-Based Derivatives and Their Molecular Docking Studies

**DOI:** 10.3390/molecules22061005

**Published:** 2017-06-17

**Authors:** Huey Chong Kwong, Siau Hui Mah, Tze Shyang Chia, Ching Kheng Quah, Gin Keat Lim, C. S. Chidan Kumar

**Affiliations:** 1 School of Chemical Sciences, Universiti Sains Malaysia, Penang 11800 USM, Malaysia; stonekg01@gmail.com (H.C.K.); limgk@usm.my (G.K.L.); 2 School of Biosciences, Taylor’s University, Lakeside Campus, 47500 Subang Jaya, Selangor, Malaysia; 3 X-ray Crystallography Unit, School of Physics, Universiti Sains Malaysia, Penang 11800 USM, Malaysia; chiatzeshyang@hotmail.com (T.S.C.); ckquah@usm.my (C.K.Q.); 4 Department of Engineering Chemistry, Vidya Vikas Institute of Engineering & Technology, Visvesvaraya Technological University, Alanahalli, Mysuru 570028, Karnataka, India; chidankumar@gmail.com

**Keywords:** adamantane, acetylcholinesterase, butyrylcholinesterase, molecular docking

## Abstract

Adamantyl-based compounds are clinically important for the treatments of type 2 diabetes and for their antiviral abilities, while many more are under development for other pharmaceutical uses. This study focused on the acetylcholinesterase (AChE) and butyrylcholinesterase (BChE) inhibitory activities of adamantyl-based ester derivatives with various substituents on the phenyl ring using Ellman’s colorimetric method. Compound **2e** with a 2,4-dichloro electron-withdrawing substituent on the phenyl ring exhibited the strongest inhibition effect against AChE, with an IC_50_ value of 77.15 µM. Overall, the adamantyl-based ester with the mono-substituent at position 3 of the phenyl ring exhibited good AChE inhibition effects with an ascending order for the substituents: Cl < NO_2_ < CH_3_ < OCH_3_. Furthermore, compounds with electron-withdrawing groups (Cl and NO_2_) substituted at position 3 on their phenyl rings demonstrated stronger AChE inhibition effects, in comparison to their respective positional isomers. On the other hand, compound **2j** with a 3-methoxyphenyl ring showed the highest inhibition effect against BChE, with an IC_50_ value of 223.30 µM. Molecular docking analyses were conducted for potential AChE and BChE inhibitors, and the results demonstrated that the peripheral anionic sites of target proteins were predominant binding sites for these compounds through hydrogen bonds and halogen interactions instead of hydrophobic interactions in the catalytic active site.

## 1. Introduction

Alzheimer’s disease (AD) is a degenerative brain disease resulting in a decline in memory, language, problem-solving, and cognitive skills, which affects a person’s ability to perform daily activities. Additionally, AD can be fatal after a prolonged period of struggle and suffering [1]. Although the intrinsic nature of the pathophysiology of AD is still unclear, a “cholinergic hypothesis” was developed from the study, targeted on people at an advanced age as well as AD patients’ basal forebrain and rostral forebrain cholinergic pathways [2]. Based on the cholinergic hypothesis, a cholinesterase inhibitor is used to enhance cognitive functions and slow down AD progression, and involves symptomatic treatment [3]. There are a limited number of drugs used as cholinesterase inhibitors in AD treatment; examples are physostigmine and galantamine [4], which originate from plants, in addition to propidium iodide [5], tacrine [6], donepezil [7], and rivastigmine [8], which are synthetic drugs.

Adamantane is a bulky diamondoid with a chemical formula of C_10_H_16_. Adamantane was first suggested by H. Decker in 1924, before it was discovered in petroleum by S. landa, V. Machaced, and M. Mzoured in 1932 using fractional distillation [9]. A decade later, V. Prelog (1941) successfully synthesized adamantane using Meerwein’s ester with a five-stage procedure [10]. Plukenetione A is the first natural product bearing an adamantane framework isolated from a plant source, namely *Clusia plukenetii* (Guttiferae), in 1966 [11]. It was found to inhibit the enzymatic activities of both topoisomerase I and DNA polymerase [12]. Furthermore, adamantan-1-amine (or amantadine) was the first adamantyl-based compound that was established as a treatment for influenza A infections, as well as to alleviate a number of symptoms of Parkinson’s disease in 1979 [13]. Additionally, the discovery of (*RS*)-1-(adamantyl)ethanamine (or rimantadine) gives a more effective prophylaxis and treatment of influenza A infections with reduced side effects [14]. Nowadays, its derivatives are applied clinically in the treatment of acne vulgaris [15], for antivirals [16,17,18,19,20], for type 2 diabetes [21,22,23], and also for AD [24].

Inspired by the diverse bioactivities of adamantane-containing molecules, herein the AChE and BChE inhibitory activities of 18 adamantyl-based ester derivatives with varying substituents were evaluated using Ellman’s colorimetric test. As reported previously, the structure of the 18 adamantyl-based ester derivatives were confirmed by NMR, FTIR, MS and single-crystal X-ray diffraction [25]. In this study, we also investigated the in silico binding mode of the proposed ligands in AChE and BChE enzymes, in comparison with tacrine as a reference, and the molecular docking procedure was validated using tacrine as the native ligand. There are two major binding sites in cholinesterase, namely the catalytic anionic site (CAS) and the peripheral anionic site (PAS). Since the inhibitors of acetylcholine bind to either CAS (tacrine, galantamine, rivastigmine), PAS (propidium iodide), or to both (donepezil), we examined the interaction of the potent inhibitors with the whole binding sites (including both CAS and PAS) through molecular docking simulations. The Genetic Optimization for Ligand Docking (GOLD) methodology was paired with a Piecewise Linear Potential (PLP) scoring function to generate the best putative pose and conformation for all subjected inhibitors.

## 2. Results and Discussion

Eighteen adamantyl-based ester derivatives (**2a**–**r**) were synthesized, and their structures were confirmed wit H-NMR, FTIR, MS, and single-crystal X-ray diffraction, as reported previously [18]. All compounds **2a**–**r** were examined for their AChE and BChE inhibitory activities by Ellman’s assay [26], and their IC_50_ values were tabulated in Table 1. Adamantyl-based ester derivatives with unsubstituted phenyl (**2a**) and pyridine (**2r**) rings were synthesized as reference compounds to accentuate the electronic effects of different substituents attached to the phenyl ring, including electron-donating (CH_3_, OCH_3_ and NH_2_) and electron-withdrawing (Cl and NO_2_) groups, upon their cholinesterase inhibitory activities. Compounds **2a** and **2r** showed low inhibition effects towards both AChE and BChE up to a concentration of 1000 µM. Similarly, compounds **2o**, **2p** and **2q** that bore strong electron-donating (NH_2_) substituents were inactive against these cholinesterase enzymes.

**Figure 1:**
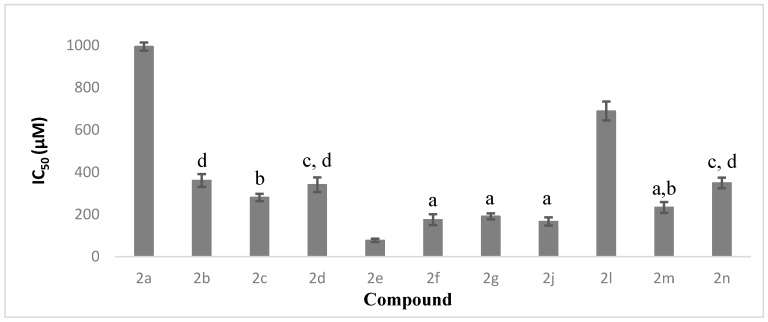
The percentage of AChE inhibition as means ± SD (*n* = 3). Means with different alphabet letters are significantly different (*p* < 0.05).

For AChE inhibition effects, compounds **2f** and **2g** with moderate electron-donating moiety and 2-methyl and 3-methyl substitutions on their phenyl rings exhibited a moderate effect, with IC_50_ values of 174.98 and 190.99 µM, respectively. The inhibition effect dropped dramatically with the methyl group substituted at position 4 on the phenyl ring, as seen in compound **2h**, which was inactive up to a concentration of 1500 µM. Although compounds **2i**, **2j** and **2k** also carried electron-donating methoxy-substituents on their phenyl rings, only **2j** with a 3-methoxy substituent showed moderate inhibition effects towards AChE, with an IC_50_ value of 166.46 µM. Comparing compounds **2f**, **2g** and **2j**, these showed no statistical difference towards their AChE inhibition activities, as shown in Figure 1.

In contrast, compounds bearing electron-withdrawing substituents on the phenyl ring, such as chlorine and nitro groups, showed relatively lower AChE inhibitory activity. As evidence, compounds **2b**, **2c** and **2d**, which had chlorine substituents on their phenyl rings at positions 2, 3 and 4, respectively, showed a range of IC_50_ values from 280 to 360 µM. Similarly, IC_50_ values for the compounds with a nitro group attached to their phenyl rings, **2m**, **2n** and **2l**, fell in the range from 233 to 690 µM. By comparing the position of the electron-withdrawing substituent on the phenyl group, adamantyl compounds with the substituent at position 3 exhibited the strongest AChE inhibition effect, followed by those with position 4 and position 2 substituents.

Overall, compound **2e**, which bore 2,4-dichloro substituents on the phenyl ring, was the most potent AChE inhibitor among the adamantyl derivatives, with an IC_50_ value of 77.15 µM. However, this compound showed weak activities against BChE, with an IC_50_ value of 306.77 µM, which was almost 5 times lower than that of the AChE inhibition effect. Although compound **2e** showed much weaker effects in the BChE inhibition assay, this compound still existed as one of the most active compounds in this enzyme inhibition test. Among the derivatives, compound **2j** exhibited the strongest inhibition towards BChE, with an IC_50_ value of 223.30 µM, followed by **2e**.

The results suggest that the substances with mono-substituents at position 3 of the phenyl ring exhibited profound effects on the AChE inhibition, and these effects increased in the order of the substituent moieties: Cl < NO_2_ < CH_3_ < OCH_3_. On the other hand, the AChE inhibition effects of the compounds with mono-substituted electron-withdrawing groups (Cl and NO_2_) on their phenyl rings increased in the order of substituents: position 2 < position 4 < position 3. A similar effect of positional substitution was also observed in 2-(2-(4-benzylpiperazin-1-yl)ethyl)isoindoline-1,3-dione derivatives [27]. Compound **2e**, with two chlorine groups substituted at positions 2 and 4, was far more potent in the AChE inhibition test compared to other mono-chloro-substituted derivatives. In fact, these compounds exerted less potency toward BChE inhibition, and encompassing an electron-donating group (methoxyl) at position 3 of the phenyl ring did increase the BChE inhibitory activity effectively. In other words, the removal of a functional group or the addition of a strong electron-donating group may decrease or deactivate the cholinesterase inhibition effects of adamantyl-based derivatives.

**Figure 2:**
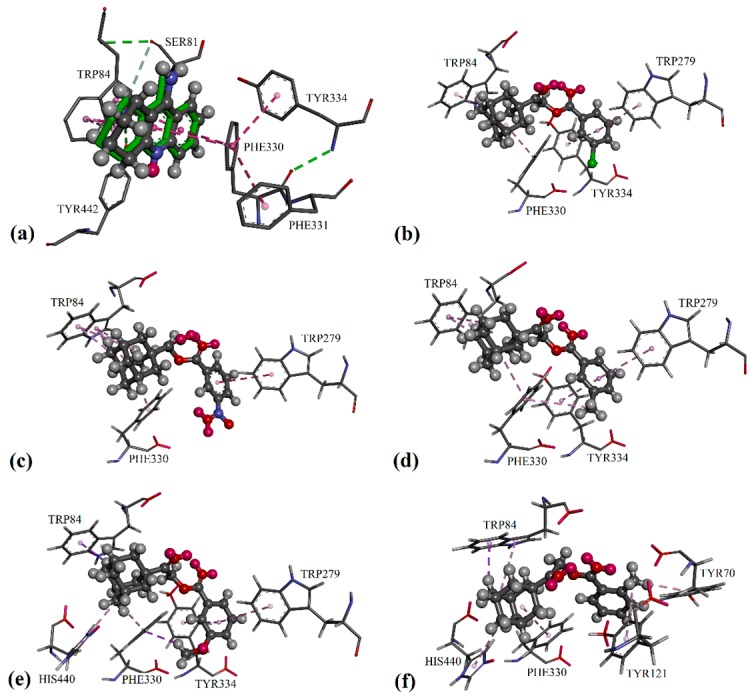
Differential validation in the GOLD package by docking the native ligands of AChE into their binding sites: (**a**) The native co-crystallized tacrine represented by green sticks; docked ligands are shown in the form of balls and sticks, colored by element. Putative binding modes of: (**b**) compound **2c**; (**c**) compound **2m**; (**d**) compound **2g**; (**e**) compound **2j**; and (**f**) compound **2f**.

Molecular docking studies were performed to provide a binding mode of adamantyl-based derivatives within the cholinesterase enzymes. Differential validation of the docking accuracy was investigated by docking native co-crystallized ligand (tacrine) into parent enzymes to compare the conformation of the best-scored pose with the bound ligand in the native crystal (see Figure 2a). The overall structure of a human’s BChE is similar to a human’s AChE, with the difference of several aromatic residues lining the gorge of AChE being replaced by hydrophobic aliphatic residue in BChE. Generally, phenylalanines (Phe) in the acyl binding pocket of AChE are replaced by leucine (Leu) and valine (Val) in BChE, which allow bulkier substrate moieties to bind more readily to BChE than to AChE [28]. However, our recent cholinesterase enzymes inhibitory study showed that bulky adamantyl-based ester derivatives exhibited a greater AChE inhibitory when compared to BChE.

**Figure 3:**
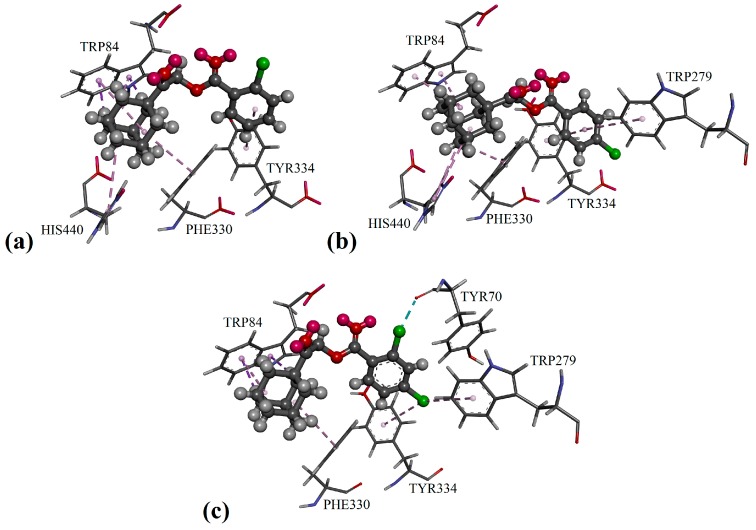
Putative binding modes in AChE enzymes of: (**a**) compound **2b**; (**b**) compound **2d**; and (**c**) compound **2e**.

In the protein crystal structure of 1ACJ, the inhibitor tacrine was bound to the catalytic active site of the protein AChE through π/π interactions, involving residues Trp84 and Phe330, as seen in Figure 2a. Similarly, adamantyl moiety in all potent substances was able to bind at the catalytic active site via alkyl/π interactions with the residues Trp84 and Phe330. Furthermore, an additional alkyl/π interaction with residue His440 was observed for compounds **2b**, **2d**, **2f** and **2j**. For those compounds with position 3 substituents on their phenyl rings (**2c**, **2g**, **2j** and **2m**), their phenyl rings tended to form π/π interactions with residues Trp279 and Tyr334 at the peripheral anionic site [29]. For compounds **2c** and **2m**, electron-withdrawing substituents on their phenyl rings failed to bind with other residues at the catalytic active site, which led to a decrease in enzyme inhibition activities (see Figure 2b,c). Conversely, the electron-donating substituent present in compounds **2g** and **2j** managed to form an extra C–H/π interaction with residue Phe330 (Figure 3d,e), and resulted in stronger inhibition effects.

**Figure 4:**
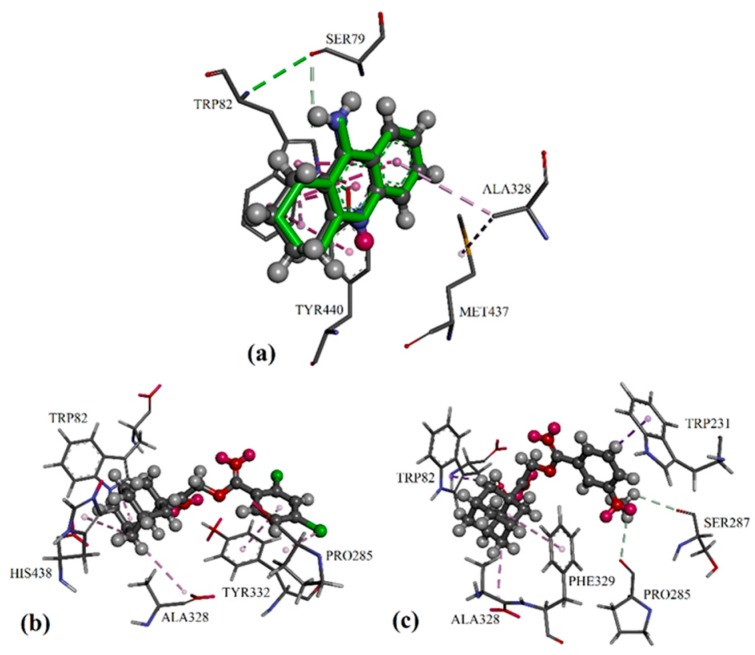
Differential validation in the GOLD package by docking the native ligands of BChE into their binding sites: (**a**) The native co-crystallized tacrine is represented by green sticks, while the docked ligands are shown in the form of balls and sticks, colored by element. Putative binding modes of: (**b**) compound **2e** in BChE, and (**c**) active compound **2j** in BChE.

Besides the π/π interaction formed with the compounds **2c**, **2g**, **2j** and **2m**, the 2-methyl substituent in compound **2f** was able to bind with residues Tyr70 and Tyr121 (see Figure 2f) in the adjacent peripheral anionic site and procure comparable inhibitory activity to compounds **2g** and **2j**. As shown in Figure 2b and Figure 3a,b, the phenyl ring of mono-chloro-substituted compounds **2b**, **2c** and **2d** was favored to bind with residues Trp279 and Tyr334 at the peripheral anionic site through π/π interactions. Furthermore, the halogen atoms of the di-chloro-substituted compound **2e** were capable of binding with residues Trp279, Tyr334 and Tyr70 via halogen and halogen/π interactions, as seen in Figure 4c, therefore exhibiting stronger inhibition effects than those of **2b** and **2d** by threefold.

For the BChE molecular docking study with a protein crystal structure of 4BDS, the inhibitor tacrine was bound to the catalytic active site of the protein BChE with alkyl/π and π/π interactions, involving residues Trp82 and Ala328, as seen in Figure 4a. Similar to AChE, adamantyl moiety of the potent compounds was able to bound at the catalytic active site via alkyl/π interactions with residues Trp82 and Ala328 in BChE, while additional alkyl/π interactions with residues His438 or Phe329 were observed for compounds **2e** and **2j**, respectively. For compound **2e**, its di-chloro-substituted phenyl ring formed π/π and halogen/π interactions with residues Tyr332 and Pro285 in both the peripheral anionic site and the acyl pocket, resulting in an increase of its BChE inhibition effect (see Figure 4b). In contrast, the 3-methoxy-substituted phenyl ring in compound **2j** was able to form intermolecular C–H/O hydrogen bonds with Pro285 of the acyl pocket and Ser287 of the peripheral anionic site (see Figure 4c). The additional C–H/π interactions with residue Trp231 led to stronger BChE inhibition effects compared to **2e**.

As a summary, the molecular docking results showed that the compounds with adamantyl moiety were able to bind with the catalytic active site via alkyl/π interactions (Trp84 and Phe330 in AChE; Trp82 and Ala328 in BChE) by utilizing the hydrophobic feature of the adamantyl ring. The finding reveals that AChE inhibition effects are attributed to the di-chloro-substituted rings, which are able to bind with residues Trp279, Tyr334 and Tyr70 at the peripheral anionic site through halogen and halogen/π interactions. On the other hand, BChE inhibition effects are mainly contributed by C–H/O hydrogen bonds between the substituted 3-methoxyphenyl ring and the residue in both the peripheral anionic site and the acyl pocket.

## 3. Materials and Methods

Electric eel acetylcholinesterase (AChE, EC 3.1.1.7), equine serum butyrylcholinesterase (BChE, EC 3.1.1.8), 5,5’-dithio-bis-2-nitrobenzoic acid (DTNB), and sodium phosphate dibasic (Na_2_HPO_4_) were purchased from Sigma-Aldrich (St. Louis, MO, USA). *S*-butyrylthiocholine chloride (BTCC) from Sigma-Aldrich (Buchs, Switzerland), acetylthiocholine iodide (ATCI) from Sigma-Aldrich (Dorset, UK) and sodium phosphate monobasic (NaH_2_PO_4_) from Sigma-Aldrich (Munich, Germany) were purchased. Tacrine hydrochloride from Cayman Chemical Company was used as the standard drug, and absolute ethanol from Merck Emsure^®^ Germany, was used as the solvent. The reagents and solvents for the synthesis work were obtained commercially from Sigma-Aldrich Corporation (St. Louis, MO, USA) and used without any further purification.

### 3.1. Synthesis and Structural Elucidation of Adamantane

**Figure 5:**
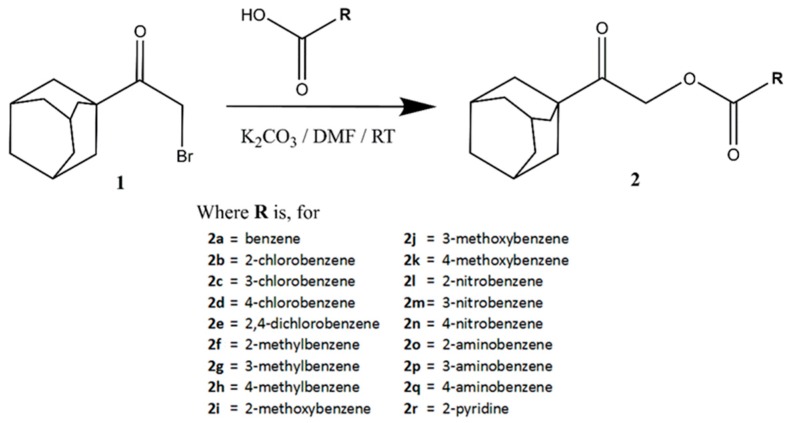
General reaction scheme for the preparation of 2-(adamantan-1-yl)-2-oxoethyl benzoates, **2a**–**q**, and 2-(adamantan-1-yl)-2-oxoethyl-2-pyridinecarboxylate, **2r**.

General synthetic routes for 2-(adamantan-1-yl)-2-oxoethyl benzoates, **2a**–**q**, and 2-(adamantan-1-yl)-2-oxoethyl-2-pyridinecarboxylate, **2r**, are shown in Figure 5. Synthesis methods, spectroscopy analyses, and single-crystal X-ray diffraction studies were described in detail in the previous report [25].

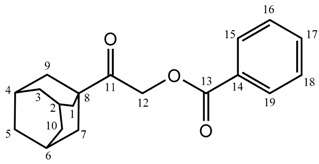



*2-(Adamantan-1-yl)-2-oxoethyl benzoate* (**2a**): Yield: 80%; m.p.: 101–103 °C; FT-IR (ATR (solid) cm^−1^): 2917, 2850 (C–H, ν), 1709 (C=O, ν), 1602, 1413 (Ar, C–H, ν), 1277, 1120 (C–O, ν), 706 (C–H, ω); ^1^H-NMR (500 MHz, CDCl_3_): δ ppm 8.095–8.081 (d, 2H, *J* = 7.5 Hz, ^15^CH, ^19^CH), 7.579–7.549 (t, 1H, *J* = 7.5 Hz, ^17^CH), 7.455–7.424 (t, 2H, *J* = 7.5 Hz, ^16^CH, ^18^CH), 5.096 (s, 2H, ^12^CH_2_), 2.079 (br-s, 3H, ^2^CH, ^4^CH, ^6^CH), 1.935–1.930 (br-d, 6H, ^1^CH_2_, ^7^CH_2_, ^9^CH_2_), 1.796–1.719 (br-q, 6H, ^3^CH_2_, ^5^CH_2_, ^10^CH_2_); ^13^C-NMR (125 MHz, CDCl_3_): δ ppm 207.16 (C^11^), 166.04 (C^13^), 133.19 (C^17^), 129.87 (C^15^, C^19^), 129.58 (C^14^), 128.37 (C^16^, C^18^), 64.94 (C^12^), 45.39 (C^8^), 38.02 (C^1^, C^7^, C^9^), 36.47 (C^3^, C^5^, C^10^), 27.79 (C^2^, C^4^, C^6^); GC-MS (EI) *m/z*: 298 (M+).

*2-(Adamantan-1-yl)-2-oxoethyl 2-chlorobenzoate* (**2b**): Yield: 83%; m.p.: 105–107 °C; FT-IR (ATR (solid) cm^−1^): 2904, 2850 (C–H, ν), 1709 (C=O, ν), 1590, 1416 (Ar, C–H, ν), 1247, 1024 (C–O, ν), 742 (C–Cl, ν); ^1^H-NMR (500 MHz, CDCl_3_): δ ppm 8.008–7.992 (d, 1H, *J* = 8.0 Hz, ^19^CH), 7.468–7.452 (d, 1H, *J* = 8.0 Hz, ^16^CH), 7.446–7.416 (t, 1H, *J* = 8.0 Hz, ^18^CH), 7.344–7.315 (t, 1H, *J* = 8.0 Hz, ^17^CH), 5.115 (s, 2H, ^12^CH_2_), 2.085 (br-s, 3H, ^2^CH, ^4^CH, ^6^CH), 1.934–1.929 (br-d, 6H, ^1^CH_2_, ^7^CH_2_, ^9^CH_2_), 1.798–1.719 (br-q, 6H, ^3^CH_2_, ^5^CH_2_, ^10^CH_2_); ^13^C-NMR (125 MHz, CDCl_3_): δ ppm 206.98 (C^11^), 164.89 (C^13^), 133.98 (C^15^), 132.84 (C^17^), 131.95 (C^19^), 131.03 (C^16^), 129.35 (C^14^), 126.62 (C^18^), 65.18(C^12^), 45.38 (C^8^), 37.99 (C^1^, C^7^, C^9^), 36.44 (C^3^, C^5^, C^10^), 27.75 (C^2^, C^4^, C^6^); GC-MS (EI) *m/z*: 332 (M+).

*2-(Adamantan-1-yl)-2-oxoethyl 3-chlorobenzoate* (**2c**): Yield: 73%; m.p.: 130–132 °C; FT-IR (ATR (solid) cm^−1^): 2911, 2850 (C–H, ν), 1718 (C=O, ν), 1571, 1442 (Ar, C–H, ν), 1295, 1253, 1130 (C–O, ν), 745 (C–Cl, ν); ^1^H-NMR (500 MHz, CDCl_3_): δ ppm 8.070 (s, 1H, ^15^CH), 7.978–7.962 (d, 1H, *J* = 7.8 Hz, ^17^CH), 7.554–7.538 (d, 1H, *J* = 7.8 Hz, ^19^CH), 7.407–7.376 (t, 1H, *J* = 7.8 Hz, ^18^CH), 5.107 (s, 2H, ^12^CH_2_), 2.086 (br-s, 3H, ^2^CH, ^4^CH, ^6^CH), 1.928–1.925 (br-d, 6H, ^1^CH_2_, ^7^CH_2_, ^9^CH_2_), 1.800–1.719 (br-q, 6H, ^3^CH_2_, ^5^CH_2_, ^10^CH_2_); ^13^C-NMR (125 MHz, CDCl_3_): δ ppm 206.94 (C^11^), 164.88 (C^13^), 134.56 (C^16^), 133.27 (C^17^), 131.27 (C^14^), 129.95 (C^18^), 129.73 (C^15^), 128.01 (C^19^), 65.23(C^12^), 45.37 (C^8^), 37.98 (C^1^, C^7^, C^9^), 36.43 (C^3^, C^5^, C^10^), 27.74 (C^2^, C^4^, C^6^); GC-MS (EI) *m/z*: 332 (M+).

*2-(Adamantan-1-yl)-2-oxoethyl 4-chlorobenzoate* (**2d**): Yield: 75%; m.p.: 138–140 °C; FT-IR (ATR (solid) cm^−1^): 2910, 2851 (C–H, ν), 1723 (C=O, ν), 1593, 1421 (Ar, C–H, ν), 1269, 1119 (C–O, ν), 752 (C–Cl, ν); ^1^H-NMR (500 MHz, CDCl_3_): δ ppm 8.029–8.011 (d, 2H, *J* = 8.7 Hz, ^16^CH, ^18^CH), 7.429–7.412 (d, 2H, *J* = 8.7 Hz, ^15^CH, ^19^CH), 5.093 (s, 2H, ^12^CH_2_), 2.082 (br-s, 3H, ^2^CH, ^4^CH, ^6^CH), 1.926–1.921 (br-d, 6H, ^1^CH_2_, ^7^CH_2_, ^9^CH_2_), 1.797–1.717 (br-q, 6H, ^3^CH_2_, ^5^CH_2_, ^10^CH_2_); ^13^C-NMR (125 MHz, CDCl_3_): δ ppm 207.07 (C^11^), 165.19 (C^13^), 139.71 (C^17^), 131.27 (C^15^, C^19^), 128.76 (C^16^, C^18^), 128.01 (C^14^), 65.11(C^12^), 45.37 (C^8^), 37.99 (C^1^, C^7^, C^9^), 36.43 (C^3^, C^5^, C^10^), 27.75 (C^2^, C^4^, C^6^); GC-MS (EI) *m/z*: 332 (M+).

*2-(Adamantan-1-yl)-2-oxoethyl 2,4-dichlorobenzoate* (**2e**): Yield: 85%; m.p.: 122–124 °C; FT-IR (ATR (solid) cm^−1^): 2912, 2850 (C–H, ν), 1711 (C=O, ν), 1583, 1417 (Ar, C–H, ν), 1244, 1129, 1098, 1023 (C–O, ν), 829 (C–Cl, ν); ^1^H-NMR (500 MHz, CDCl_3_): δ ppm 7.976–7.959 (d, 1H, *J* = 8.5 Hz, ^19^CH), 7.484 (s, 1H, ^16^CH), 7.329–7.312 (d, 1H, *J* = 8.5 Hz, ^18^CH), 5.105 (s, 2H, ^12^CH_2_), 2.085 (br-s, 3H, ^2^CH, ^4^CH, ^6^CH), 1.925–1.920 (br-d, 6H, ^1^CH_2_, ^7^CH_2_, ^9^CH_2_), 1.799–1.717 (br-q, 6H, ^3^CH_2_, ^5^CH_2_, ^10^CH_2_); ^13^C-NMR (125 MHz, CDCl_3_): δ ppm 206.86 (C^11^), 164.01 (C^13^), 138.66 (C^15^), 135.20 (C^17^), 133.00 (C^19^), 131.00 (C^16^), 127.62 (C^14^), 127.06 (C^18^), 65.30 (C^12^), 45.38 (C^8^), 37.98 (C^1^, C^7^, C^9^), 36.42 (C^3^, C^5^, C^10^), 27.73 (C^2^, C^4^, C^6^); GC-MS (EI) *m/z*: 366 (M+).

*2-(Adamantan-1-yl)-2-oxoethyl 2-methylbenzoate* (**2f**): Yield: 83%; m.p.: 94–96 °C; FT-IR (ATR (solid) cm^−1^): 2905, 2851 (C–H, ν), 1708 (C=O, ν), 1602, 1415 (Ar, C–H, ν), 1253, 1026 (C–O, ν), 733 (C–H, ω); ^1^H-NMR (500 MHz, CDCl_3_): δ ppm 8.018–8.002 (d, 1H, *J* = 8.0 Hz, ^19^CH), 7.421–7.391 (t, 1H, *J* = 8.0 Hz, ^17^CH), 7.252–7.235 (m, 2H, ^16^CH, ^18^CH), 5.087 (s, 2H, ^12^CH_2_), 2.607 (s, 3H, ^20^CH_3_), 2.085 (br-s, 3H, ^2^CH, ^4^CH, ^6^CH), 1.940–1.935 (br-d, 6H, ^1^CH_2_, ^7^CH_2_, ^9^CH_2_), 1.799–1.721 (br-q, 6H, ^3^CH_2_, ^5^CH_2_, ^10^CH_2_); ^13^C-NMR (125 MHz, CDCl_3_): δ ppm 207.41 (C^11^), 166.98 (C^13^), 140.43 (C^15^), 132.19 (C^17^), 131.58 (C^16^), 130.89 (C^19^), 129.07 (C^14^), 125.72 (C^18^), 64.74 (C^12^), 45.37 (C^8^), 38.03 (C^1^, C^7^, C^9^), 36.47 (C^3^, C^5^, C^10^), 27.78 (C^2^, C^4^, C^6^), 21.60 (C^20^); GC-MS (EI) *m/z*: 312 (M+).

*2-(Adamantan-1-yl)-2-oxoethyl 3-methylbenzoate* (**2g**): Yield: 75%; m.p.: 99–101 °C; FT-IR (ATR (solid) cm^−1^): 2902, 2848 (C–H, ν), 1724 (C=O, ν), 1588, 1421, (Ar, C–H, ν), 1302, 1279, 1195, 1109 (C–O, ν), 740 (C–H, ω); ^1^H-NMR (500 MHz, CDCl_3_): δ ppm 7.909 (s, 1H, ^15^CH) 7.894–7.879 (d, 2H, *J* = 7.6 Hz, ^19^CH), 7.388–7.372 (d, 1H, *J* = 7.6 Hz, ^17^CH), 7.345–7.315 (t, 1H, *J* = 7.6 Hz, ^18^CH), 5.093 (s, 2H, ^12^CH_2_), 2.397 (s, 3H, ^20^CH_3_), 2.083 (br-s, 3H, ^2^CH, ^4^CH, ^6^CH), 1.936–1.931 (br-d, 6H, ^1^CH_2_, ^7^CH_2_, ^9^CH_2_), 1.798–1.721 (br-q, 6H, ^3^CH_2_, ^5^CH_2_, ^10^CH_2_); ^13^C-NMR (125 MHz, CDCl_3_): δ ppm 207.32 (C^11^), 166.25 (C^13^), 138.17 (C^16^), 134.00 (C^15^), 130.39 (C^17^), 129.44 (C^14^), 128.29 (C^18^), 127.04 (C^19^), 64.91 (C^12^), 45.39 (C^8^), 38.01 (C^1^, C^7^, C^9^), 36.47 (C^3^, C^5^, C^10^), 27.78 (C^2^, C^4^, C^6^), 21.26 (C^20^); GC-MS (EI) *m/z*: 312 (M+).

*2-(Adamantan-1-yl)-2-oxoethyl 4-methylbenzoate* (**2h**): Yield: 81%; m.p.: 140–142 °C; FT-IR (ATR (solid) cm^−1^): 2902, 2853 (C–H, ν), 1714 (C=O, ν), 1610, 1418, (Ar, C–H, ν), 1258, 1115, (C–O, ν), 747 (C–H, ω); ^1^H-NMR (500 MHz, CDCl_3_): δ ppm 7.984–7.968 (d, 2H, *J* = 8.1 Hz, ^15^CH, ^19^CH), 7.249–7.233 (d, 2H, *J* = 8.1 Hz, ^16^CH, ^18^CH), 5.080 (s, 2H, ^12^CH_2_), 2.410 (s, 3H, ^20^CH_3_), 2.080 (br-s, 3H, ^2^CH, ^4^CH, ^6^CH), 1.934–1.929 (br-d, 6H, ^1^CH_2_, ^7^CH_2_, ^9^CH_2_), 1.795–1.718 (br-q, 6H, ^3^CH_2_, ^5^CH_2_, ^10^CH_2_); ^13^C-NMR (125 MHz, CDCl_3_): δ ppm 207.37 (C^11^), 166.12 (C^13^), 143.93 (C^17^), 129.92 (C^15^, C^19^), 129.10 (C^16^, C^18^), 126.80 (C^14^), 64.82 (C^12^), 45.39 (C^8^), 38.01 (C^1^, C^7^, C^9^), 36.47 (C^3^, C^5^, C^10^), 27.79 (C^2^, C^4^, C^6^), 21.70 (C^20^); GC-MS (EI) *m/z*: 312 (M+).

*2-(Adamantan-1-yl)-2-oxoethyl 2-methoxybenzoate* (**2i**): Yield: 80%; m.p.: 89–91 °C; FT-IR (ATR (solid) cm^−1^): 2902, 2851 (C–H, ν), 1702 (C=O, ν), 1599, 1442 (Ar, C–H, ν), 1248, 1096, 1020 (C–O, ν), 758 (C–H, ω); ^1^H-NMR (500 MHz, CDCl_3_): δ ppm 7.966–7.950 (d, 1H, *J* = 7.8 Hz, ^19^CH), 7.500–7.468 (t, 1H, *J* = 7.8 Hz, ^17^CH), 7.005–6.972 (t, 2H, *J* = 7.8 Hz, ^16^CH, ^18^CH), 5.071 (s, 2H, ^12^CH_2_), 3.907 (s, 3H, ^20^CH_3_), 2.074 (br-s, 3H, ^2^CH, ^4^CH, ^6^CH), 1.931–1.926 (br-d, 6H, ^1^CH_2_, ^7^CH_2_, ^9^CH_2_), 1.791–1.714 (br-q, 6H, ^3^CH_2_, ^5^CH_2_, ^10^CH_2_); ^13^C-NMR (125 MHz, CDCl_3_): δ ppm 207.41 (C^11^), 165.23 (C^13^), 159.52 (C^15^), 133.90 (C^17^), 132.21 (C^19^), 120.17 (C^18^), 119.19 (C^14^), 111.99 (C^16^), 64.72 (C^12^), 56.03 (C^20^), 45.38 (C^8^), 38.01 (C^1^, C^7^, C^9^), 36.48 (C^3^, C^5^, C^10^), 27.79 (C^2^, C^4^, C^6^); GC-MS (EI) *m/z*: 328 (M+).

*2-(Adamantan-1-yl)-2-oxoethyl 3-methoxybenzoate* (**2j**): Yield: 74%; m.p.: 157–159 °C; FT-IR (ATR (solid) cm^−1^): 2926, 2853 (C–H, ν), 1711 (C=O, ν), 1584, 1489 (Ar, C–H, ν), 1288, 1221, 1029 (C–O, ν), 759 (C–H, ω); ^1^H-NMR (500 MHz, CDCl_3_): δ ppm 7.700–7.685 (d, 1H, *J* = 7.9 Hz, ^19^CH), 7.598 (s, 1H, ^15^CH), 7.365–7.333 (t, 1H, *J* = 7.9 Hz, ^18^CH), 7.127–7.110 (d, 1H, *J* = 7.9 Hz, ^17^CH), 5.093 (s, 2H, ^12^CH_2_), 3.846 (s, 3H, ^20^CH_3_), 2.083 (br-s, 3H, ^2^CH, ^4^CH, ^6^CH), 1.936–1.931 (br-d, 6H, ^1^CH_2_, ^7^CH_2_, ^9^CH_2_), 1.798–1.721 (br-q, 6H, ^3^CH_2_, ^5^CH_2_, ^10^CH_2_); ^13^C-NMR (125 MHz, CDCl_3_): δ ppm 207.18 (C^11^), 165.96 (C^13^), 159.56 (C^16^), 130.79 (C^14^), 129.42 (C^18^), 122.35 (C^19^), 120.04 (C^17^), 114.05 (C^15^), 65.04 (C^12^), 55.44 (C^20^), 45.39 (C^8^), 38.01 (C^1^, C^7^, C^9^), 36.46 (C^3^, C^5^, C^10^), 27.77 (C^2^, C^4^, C^6^); GC-MS (EI) *m/z*: 328 (M+).

*2-(Adamantan-1-yl)-2-oxoethyl 4-methoxybenzoate* (**2k**): Yield: 80%; m.p.: 117–119 °C; FT-IR (ATR (solid) cm^−1^): 2903, 2851 (C–H, ν), 1710 (C=O, ν), 1605, 1417 (Ar, C–H, ν), 1253, 1167, 1028 (C–O, ν), 767 (C–H, ω); ^1^H-NMR (500 MHz, CDCl_3_): δ ppm 8.053–8.035 (d, 2H, *J* = 9.0 Hz, ^15^CH, ^19^CH), 6.932–6.914 (d, 2H, *J* = 9.0 Hz, ^16^CH, ^18^CH), 5.069 (s, 2H, ^12^CH_2_), 3.863 (s, 3H, ^20^CH_3_), 2.080 (br-s, 3H, ^2^CH, ^4^CH, ^6^CH), 1.933–1.928 (br-d, 6H, ^1^CH_2_, ^7^CH_2_, ^9^CH_2_), 1.795–1.718 (br-q, 6H, ^3^CH_2_, ^5^CH_2_, ^10^CH_2_); ^13^C-NMR (125 MHz, CDCl_3_): δ ppm 207.54 (C^11^), 165.77 (C^13^), 163.61 (C^17^), 131.96 (C^15^, C^19^), 121.94 (C^14^), 113.66 (C^16^, C^18^), 64.73 (C^12^), 55.44 (C^20^), 45.39 (C^8^), 38.02 (C^1^, C^7^, C^9^), 36.47 (C^3^, C^5^, C^10^), 27.79 (C^2^, C^4^, C^6^); GC-MS (EI) *m/z*: 328 (M+).

*2-(Adamantan-1-yl)-2-oxoethyl 2-nitrobenzoate* (**2l**): Yield: 70%; m.p.: 118–120 °C; FT-IR (ATR (solid) cm^−1^): 2919, 2850 (C–H, ν), 1731 (C=O, ν), 1579, 1450 (Ar, C–H, ν), 1535, 1353 (N–O, ν), 1290, 1132, 1079 (C–O, ν), 732 (C–H, ω); ^1^H-NMR (500 MHz, CDCl_3_): δ ppm 7.970–7.954 (d, 1H, *J* = 7.7 Hz, ^16^CH), 7.935–7.920 (d, 1H, *J* = 7.7 Hz, ^19^CH), 7.726–7.695 (t, 1H, *J* = 7.7 Hz, ^18^CH), 7.663–7.631 (t, 1H, *J* = 7.7 Hz, ^17^CH), 5.123 (s, 2H, ^12^CH_2_), 2.088 (br-s, 3H, ^2^CH, ^4^CH, ^6^CH), 1.919 (br-s, 6H, ^1^CH_2_, ^7^CH_2_, ^9^CH_2_), 1.776–1.743 (br-d, 6H, ^3^CH_2_, ^5^CH_2_, ^10^CH_2_); ^13^C-NMR (125 MHz, CDCl_3_): δ ppm 206.75 (C^11^), 164.97 (C^13^), 147.74 (C^15^), 133.17 (C^17^), 131.78 (C^18^), 130.35 (C^19^), 127.46 (C^14^), 123.94 (C^16^), 65.92 (C^12^), 45.39 (C^8^), 37.95 (C^1^, C^7^, C^9^), 36.41 (C^3^, C^5^, C^10^), 27.72 (C^2^, C^4^, C^6^); GC-MS (EI) *m/z*: 343 (M+).

*2-(Adamantan-1-yl)-2-oxoethyl 3-nitrobenzoate* (**2m**): Yield: 72%; m.p.: 112–114 °C; FT-IR (ATR (solid) cm^−1^): 3092 (Ar, C–H, ν), 2917, 2851 (C–H, ν), 1727 (C=O, ν), 1631, 1421 (Ar, C–H, ν), 1533, 1347 (N–O, ν), 1297, 1261, 1131 (C–O, ν), 716 (C–H, ω); ^1^H-NMR (500 MHz, CDCl_3_): δ ppm 8.923 (s, 1H, ^15^CH), 8.449–8.432 (d, 1H, *J* = 8.0 Hz, ^17^CH), 8.417–8.402 (d, 1H, *J* = 8.0 Hz, ^19^CH), 7.685–7.653 (t, 1H, *J* = 8.0 Hz, ^18^CH), 5.167 (s, 2H, ^12^CH_2_), 2.101 (br-s, 3H, ^2^CH, ^4^CH, ^6^CH), 1.940 (br-s, 6H, ^1^CH_2_, ^7^CH_2_, ^9^CH_2_), 1.788–1.756 (br-q, 6H, ^3^CH_2_, ^5^CH_2_, ^10^CH_2_); ^13^C-NMR (125 MHz, CDCl_3_): δ ppm 206.60 (C^11^), 164.00 (C^13^), 148.31 (C^16^), 135.52 (C^19^), 131.35 (C^14^), 129.67 (C^18^), 127.66 (C^17^), 124.92 (C^15^), 65.61 (C^12^), 45.39 (C^8^), 37.99 (C^1^, C^7^, C^9^), 36.42 (C^3^, C^5^, C^10^), 27.74 (C^2^, C^4^, C^6^); GC-MS (EI) *m/z*: 343 (M+).

*2-(Adamantan-1-yl)-2-oxoethyl 4-nitrobenzoate* (**2n**):Yield: 76%; m.p.: 164–166 °C; FT-IR (ATR (solid) cm^−1^): 3112 (Ar, C–H, ν), 2905, 2854 (C–H, ν), 1723 (C=O, ν), 1606, 1422 (Ar, C–H, ν), 1530, 1344 (N–O, ν), 1283, 1118 (C–O, ν), 714 (C–H, ω); ^1^H-NMR (500 MHz, CDCl_3_): δ ppm 8.310–8.292 (d, 2H, *J* = 8.8 Hz, ^16^CH ^18^CH), 8.265–8.248 (d, 2H, *J* = 8.8 Hz, ^15^CH, ^19^CH), 5.156 (s, 2H, ^12^CH_2_), 2.100 (br-s, 3H, ^2^CH, ^4^CH, ^6^CH), 1.936 (br-d, 6H, ^1^CH_2_, ^7^CH_2_, ^9^CH_2_), 1.811–1.729 (br-q, 6H, ^3^CH_2_, ^5^CH_2_, ^10^CH_2_); ^13^C-NMR (125 MHz, CDCl_3_): δ ppm 206.61 (C^11^), 164.21 (C^13^), 150.72 (C^17^), 134.97 (C^14^), 131.00 (C^15^, C^19^), 123.56 (C^16^, C^18^), 65.61 (C^12^), 45.40 (C^8^), 37.99 (C^1^, C^7^, C^9^), 36.41 (C^3^, C^5^, C^10^), 27.73 (C^2^, C^4^, C^6^); GC-MS (EI) *m/z*: 343 (M+).

*2-(adamantan-1-yl)-2-oxoethyl 2-aminobenzoate* (**2o**): Yield: 76%; m.p.: 167–169 °C; FT-IR (ATR (solid) cm^−1^): 3497, 3373 (N–H, ν), 2902, 2848 (C–H, ν), 1695 (C=O, ν), 1613, 1418 (Ar, C–H, ν), 1581 (N–H, δ), 1243, 1104 (C–O, ν), 746 (C–H, ω); ^1^H-NMR (500 MHz, CDCl_3_): δ ppm 7.954–7.940 (d, 1H, *J* = 8.3 Hz, ^19^CH), 7.287–7.256 (t, 1H, *J* = 8.3 Hz ^17^CH), 6.667–6.639 (m, 2H, ^16^CH, ^18^CH), 5.647 (br-s, 2H, ^1^NH_2_), 5.068 (s, 2H, ^12^CH_2_), 2.083 (br-s, 3H, ^2^CH, ^4^CH, ^6^CH), 1.933–1.928 (br-d, 6H, ^1^CH_2_, ^7^CH_2_, ^9^CH_2_), 1.798–1.721 (br-q, 6H, ^3^CH_2_, ^5^CH_2_, ^10^CH_2_); ^13^C-NMR (125 MHz, CDCl_3_): δ ppm 207.64 (C^11^), 167.29 (C^13^), 150.52 (C^15^), 134.38 (C^17^), 131.63 (C^19^), 116.67 (C^18^), 116.41 (C^16^), 110.34 (C^14^), 64.46 (C^12^), 45.38 (C^8^), 38.05 (C^1^, C^7^, C^9^), 36.47 (C^3^, C^5^, C^10^), 27.79 (C^2^, C^4^, C^6^); GC-MS (EI) *m/z*: 313 (M+).

*2-(adamantan-1-yl)-2-oxoethyl 3-aminobenzoate* (**2p**): Yield: 70%; m.p.: 123–125 °C; FT-IR (ATR (solid) cm^−1^): 3465, 3347 (N–H, ν), 2902, 2849 (C–H, ν), 1700 (C=O, ν), 1631, 1461 (Ar, C–H, ν), 1603 (N–H, δ), 1248, 1113 (C–O, ν), 751 (C–H, ω); ^1^H-NMR (500 MHz, CDCl3): δ ppm 7.502–7.486 (d, 1H, *J* = 7.8 Hz, ^19^CH), 7.415 (s, 1H, ^15^CH), 7.242–7.211 (t, 1H, *J* = 7.8 Hz, ^18^CH), 6.907–6.891 (d, 1H, *J* = 7.8 Hz, ^17^CH), 5.074 (s, 2H, ^12^CH_2_), 2.080 (br-s, 3H, ^2^CH, ^4^CH, ^6^CH), 1.929–1.923 (br-d, 6H, ^1^CH_2_, ^7^CH_2_, ^9^CH_2_), 1.794–1.717 (br-q, 6H, ^3^CH_2_, ^5^CH_2_, ^10^CH_2_); ^13^C-NMR (125 MHz, CDCl3): δ ppm 207.36 (C^11^), 166.17 (C^13^), 145.98 (C^16^), 130.49 (C^14^), 129.34 (C^18^), 120.42 (C^19^), 119.99 (C^17^), 116.28 (C^15^), 64.91 (C^12^), 45.39 (C^8^), 38.00 (C^1^, C^7^, C^9^), 36.46 (C^3^, C^5^, C^10^), 27.77 (C^2^, C^4^, C^6^); GC-MS (EI) *m/z*: 313 (M+).

*2-(adamantan-1-yl)-2-oxoethyl 4-aminobenzoate* (**2q**): Yield: 76%; m.p.: 155–157 °C; FT-IR (ATR (solid) cm^−1^): 3493, 3362 (N–H, ν), 2906, 2850 (C–H, ν), 1697 (C=O, ν), 1619, 1414 (Ar, C–H, ν), 1600 (N–H, δ), 1276, 1118 (C–O, ν), 768 (C–H, ω); ^1^H-NMR (500 MHz, CDCl_3_): δ ppm 7.907–7.890 (d, 2H, *J* = 8.8 Hz, ^15^CH, ^19^CH), 6.648–6.631 (d, 2H, *J* = 8.8 Hz, ^16^CH, ^18^CH), 5.039 (s, 2H, ^12^CH_2_), 4.075 (b-s, 2H, ^1^NH_2_), 2.074 (br-s, 3H, ^2^CH, ^4^CH, ^6^CH), 1.929 (br-s, 6H, ^1^CH_2_, ^7^CH_2_, ^9^CH_2_), 1.789–1.714 (br-q, 6H, ^3^CH_2_, ^5^CH_2_, 10CH_2_); ^13^C-NMR (125 MHz, CDCl_3_): δ ppm 207.88 (C^11^), 166.07 (C^13^), 151.12 (C^17^), 132.06 (C^15^, C^19^), 118.99 (C^14^), 113.88 (C^16^, C^18^), 64.57 (C^12^), 45.42 (C^8^), 38.04 (C^1^, C^7^, C^9^), 36.50 (C^3^, C^5^, C^10^), 27.82 (C^2^, C^4^, C^6^); GC-MS (EI) *m/z*: 313 (M+).

*2-(Adamantan-1-yl)-2-oxoethyl picolinate* (**2r**): Yield: 76%; M.P.: 129–131 °C; FT-IR (ATR (solid) cm^−1^): 2903, 2849 (C–H, ν), 1707 (C=O, ν), 1582, 1419 (Ar, C–H, ν), 1304 (C–N, ν) 1242, 1128 (C–O, ν), 745 (C–H, ω); ^1^H-NMR (500 MHz, CDCl_3_): δ ppm 8.787–8.772 (d, 1H, *J* = 7.6 Hz, ^18^CH), 8.188–8.172 (d, 1H, *J* = 7.6 Hz, ^15^CH), 7.883–7.852 (t, 1H, *J* = 7.6 Hz, ^16^CH), 7.519–7.495 (q, 1H, *J* = 7.6 Hz, ^17^CH), 5.203 (s, 2H, ^12^CH_2_), 2.085 (br-s, 3H, ^2^CH, ^4^CH, ^6^CH), 1.934–1.929 (br-d, 6H, ^1^CH_2_, ^7^CH_2_, ^9^CH_2_), 1.798–1.720 (br-q, 6H, ^3^CH_2_, ^5^CH_2_, ^10^CH_2_); ^13^C-NMR (125 MHz, CDCl_3_): δ ppm 206.36 (C^11^), 164.54 (C^13^), 149.86 (C^18^), 147.41 (C^14^), 137.12 (C^16^), 127.18 (C^17^), 125.53 (C^15^), 65.70 (C^12^), 45.31 (C^8^), 37.99 (C^1^, C^7^, C^9^), 36.44 (C^3^, C^5^, C^10^), 27.75 (C^2^, C^4^, C^6^); GC-MS (EI) *m/z*: 299 (M+).

### 3.2. Cholinesterases Inhibitory Assay

AChE and BChE inhibitory activities of the compounds were determined by Ellman’s assay with minor modifications [26]. Briefly, 210 µL of a 100 mM sodium phosphate buffer (pH 8) was added to a 96-well microplate, followed by 20 µL of the compounds in ethanol and 20 µL of AChE (5.32 × 10^−3^ U). After a pre-incubation of 15 min at room temperature, 10 µL of 0.50 mM DTNB was added into each well, followed by 10 µL of 0.71 mM acetylthiocholine iodide as the substrate. The absorbance of each well was measured 10 times with 1 min intervals using the Epoch2, BioTek instrument microplate reader (Winooski, VT, USA) at 412 nm. For the BChE inhibition assay, the same procedure as described above was used, except the enzyme and substrate were replaced with BChE (6.85 × 10^3^ U) from Equine serum and 0.20 mM butyrylthiocholine chloride, respectively. Tacrine was used as a reference drug. A series of dilutions made up of 6 concentrations of compounds or the reference drug were tested to obtain the half-maximal inhibitory concentration (IC_50_). Each test was carried out in triplicate and the results were expressed as an average value. The percentage of inhibition was calculated using the following formula:

(1) $$\mathrm{Percentage}\;\mathrm{of}\;\mathrm{inhibition}=\frac{S\;\mathrm{blank}\;-S\;\mathrm{sample}}{S\;\mathrm{blank}}\;\times \;100\%$$

where **S** blank is slope of the blank reaction (without compounds or a reference standard) and **S** sample is the slope of the sample reaction (with compounds or a reference drug).

### 3.3. Statistical Analysis

The results of the cholinesterase inhibitory assay were expressed as means ± SD, and were labeled if *p* < 0.05 using ANOVA of IBM SPSS Statistics for Windows, Version 23.0 (IBM, New York, NY, USA).

### 3.4. Docking Protocol

Molecular docking was performed for the most potent inhibitors using the Genetic Optimization for Ligand Docking (GOLD) package 5.4.1 [30,31,32]. The atomic coordinates of target inhibitors were obtained directly from previously reported X-ray crystallography data [10], except for ligand **2m**, for which coordinates were constructed in the ChemBio3D workspace and were energy-minimized by MM2 force fields. The crystal structures of AChE (PDB ID: 1ACJ [33]) and BChE (PDB ID: 4BDS [34]) were isolated from *Pteronarcys californica* and *Homo sapiens*, respectively, with tacrine obtained from the Protein Data Bank ligand-protein binding space, and the conformational flexibility of the ligand inside the protein was explored by the Genetic Algorithm (GA). A spherical binding site with a radius of 15 Å was used, which lay around the binding site of the tacrine covering both the catalytic anionic and peripheral anionic sites. Runs were carried out (100 GA), and the top 100 ranked docking poses were scored using the Piecewise Linear Potential (PLP) scoring function. Default values were used for all other parameters. The intermolecular interactions of the best-scored pose of each ligand were analyzed and illustrated using Discovery Studio 4.5 software (Discovery Studio, v4.5.0.15071; San Diego, CA, USA, Accelrys Inc.; 2015).

## 4. Conclusions

Eighteen adamantyl-based ester derivatives were synthesized, and their anti-cholinesterase activities were assessed using Ellman’s assay. The AChE inhibition effects of the adamantyl-based ester derivatives increased in the order Cl < NO_2_ < CH_3_ < OCH_3_ for mono-substituents at position 3 of the phenyl ring. Moreover, those derivatives with mono-substituted electron-donating groups (Cl and NO_2_) on their phenyl rings showed an increasing inhibition effect in the order: position 2 < position 4 < position 3. As such, compound **2e**, which bore a 2,4-dichlorophenyl ring, exhibited the strongest AChE inhibition effects, while compound **2j**, with a 3-methoxyphenyl ring, was the most potent BChE inhibitor. The in silico study of the adamantyl derivatives demonstrated that the halogen interaction and hydrogen bond between the compounds and their surrounding residues in the peripheral anionic site and acyl pocket of target enzymes were predominant when compared to hydrophobic interactions in the catalytic active site.

## Figures and Tables

**Table 1:** IC_50_ values of the AChE and BChE inhibitory effects of adamantyl-based compounds.

Compounds	Substituent	AChE IC_50_ (µM)	BChE IC_50_ (µM)
**2a**	benzene	994.26 ± 19.35	>1500
**2b**	2-chlorobenzene	360.56 ± 30.05	>1500
**2c**	3-chlorobenzene	280.43 ± 17.35	971.50 ± 62.55
**2d**	4-chlorobenzene	340.52 ± 34.69	1291.99 ± 30.05
**2e**	2,4-dichlorobenzene	**77.15 ± 7.86**	306.77 ± 25.15
**2f**	2-methybenzene	174.98 ± 25.87	>1500
**2g**	3-methybenzene	190.99 ± 14.43	>1500
**2h**	4-methybenzene	>1500	>1500
**2i**	2-methoxybenzene	>1500	>1500
**2j**	3-methoxybenzene	166.46 ± 19.58	**223.30 ± 17.58**
**2k**	4-methoxybenzene	>1500	>1500
**2l**	2-nitrobenzene	689.23 ± 44.48	>1500
**2m**	3-nitrobenzene	232.98 ± 25.88	>1500
**2n**	4-nitrobenzene	349.47 ± 25.22	776.59 ± 33.63
**2o**	2-aminobenzene	>1500	>1500
**2p**	3-aminobenzene	>1500	>1500
**2q**	4-aminobenzene	>1500	>1500
**2r**	2-pyridine	>1500	>1500
Tacrine		0.21 ± 0.04	0.06 ± 0.01

Note: AChE represents Acetylcholinesterase and BChE represents Butyrylcholinesterase.
